# Cryptotanshinone from the *Salvia miltiorrhiza* Bunge Attenuates Ethanol-Induced Liver Injury by Activation of AMPK/SIRT1 and Nrf2 Signaling Pathways

**DOI:** 10.3390/ijms21010265

**Published:** 2019-12-30

**Authors:** Arulkumar Nagappan, Ji-Hyun Kim, Dae Young Jung, Myeong Ho Jung

**Affiliations:** 1 Healthy Aging Korean Medical Research Center, School of Korean Medicine, Pusan National University, Yangsan 50612, Korea; arulbiotechtnau@gmail.com (A.N.); kimji77@pusan.ac.kr (J.-H.K.); dyjung999@naver.com (D.Y.J.); 2 Division of Longevity and Biofunctional Medicine, School of Korean Medicine, Pusan National University, Yangsan 50612, Korea

**Keywords:** cryptotanshinone, AMP-activated protein kinase, nuclear factor E2-related factor 2, alcohol liver disease, cytochrome P450 2E1, sirtuin 1

## Abstract

Cryptotanshinone (CT), a diterpene that is isolated from *Salvia miltiorrhiza* Bunge, exhibits anti-cancer, anti-oxidative, anti-fibrosis, and anti-inflammatory properties. Here, we examined whether CT administration possess a hepatoprotective effect on chronic ethanol-induced liver injury. We established a chronic alcohol feeding mouse model while using C57BL/6 mice, and examined the liver sections with hematoxylin-eosin (H&E) and Oil Red O (ORO) staining. Further, we analyzed the lipogenesis, fatty acid oxidation, oxidative stress, and inflammation genes by using quantitative polymerase chain reaction (qPCR) and immunoblotting in in vivo, and in vitro while using HepG2 and AML-12 cells. CT treatment significantly ameliorated ethanol-promoted hepatic steatosis, which was consistent with the decreased hepatic triglyceride levels. Interestingly, CT activated the phosphorylation of AMP-activated protein kinase (*AMPK*), sirtuin 1 (*SIRT1*), and nuclear factor E2-related factor 2 (*Nrf2*) proteins. Importantly, compound C (*AMPK* inhibitor) significantly blocked the CT-mediated reduction in TG accumulation, but not Ex52735 (*SIRT1* inhibitor), which suggested that CT countering ethanol-promoted hepatic steatosis is mediated by *AMPK* activation. Furthermore, CT significantly inhibited cytochrome P450 2E1 (*CYP2E1*) and enhanced both the expression of antioxidant genes and hepatic glutathione levels. Finally, CT inhibited the ethanol-induced inflammation in ethanol-fed mice and HepG2 cells. Overall, CT exhibits a hepatoprotective effect against ethanol-induced liver injury by the inhibition of lipogenesis, oxidative stress, and inflammation through the activation of *AMPK/SIRT1* and *Nrf2* and the inhibition of *CYP2E1*. Therefore, CT could be an effective therapeutic agent for treating ethanol-induced liver injury.

## 1. Introduction

The dried roots of *Salvia miltiorrhiza* Bunge, called Danshen, has been used in Chinese folk medicine for over a thousand years to treat various disorders, including heart disease, liver diseases, haematological abnormalities, cerebrovascular disease, haemorrhage, menstrual disorders, miscarriage, as well as oedema and insomnia [1,2,3,4]. Recent studies have demonstrated that *S. miltiorrhiza* ameliorates carbon tetrachloride (CCl_4_)-induced liver fibrosis in vivo and in vitro [5]. Tanshinone IIA, dihydrotanshinone I, tanshinone I, and cryptotanshinone are the major abietane diterpene isolates from the root of *S. miltiorrhiza* all of which are potent antioxidants that suppress lipid peroxidation and remedy for various metabolic disorders [6,7]. Among these, cryptotanshinone (CT) is reported to have various biological functions, such as anti-cancer, anti-inflammatory, and anti-oxidative activities [8,9]. Specifically, CT was able to effectively inhibit lipopolysaccharide (LPS)-triggered Toll-like receptor 4 (*TLR4*) signaling and nuclear factor-kappa B (*NF-κB*) downstream pathways in murine macrophage RAW 264.7 [8]. In addition, CT ameliorates ethanol-induced hepatotoxicity by blocking hepatic cell death and fatty acid synthesis in primary rat hepatocytes [10]. However, the protective effects of CT against alcohol-induced fatty liver, and the exact underlying molecular mechanisms have not yet been reported. Hence, studying the protective effect of CT against alcoholic liver disease (ALD) might uncover the precise protective mechanism and provide a therapeutic potential for ALD treatment.

Alcoholism leads to alcoholic liver disease (ALD), which is a major cause of alcohol-related morbidity worldwide [11]. According to the WHO, alcohol consumption caused more than three-million deaths worldwide in 2016. The mortality rate due to alcohol abuse is higher than that of tuberculosis, HIV/AIDS, and diabetes. Fatty liver formation is an early stage of the ALD, which can evolve into advanced conditions, such as steatohepatitis, cirrhosis, fibrosis, and hepatocarcinoma [12,13,14]. There are multiple mechanisms that are associated with alcoholic fatty liver, including augmentation of lipogenesis, lipid peroxidation, oxidative stress, and the inhibition of fatty acid oxidation [12,15]. Thus, the inhibition of triglycerides (TG) and regulation of lipid metabolism could be a feasible remedy for ALD in terms of clinical outcomes and economic feasibility.

The AMP-activated protein kinase (*AMPK*) is the main sensor of cellular energy status [16], and it plays a crucial role in glucose and lipid metabolism [17]. Many studies suggest that the activation of *AMPK* could increase fatty acid oxidation and decrease lipogenesis [15,18,19,20]. *AMPK* can also activate sirtuin 1 (*SIRT1*), a nicotinamide adenosine dinucleotide (NAD)-dependent deacetylase, by increasing the substrate for *SIRT1* activity that is NAD+ levels [21], and *SIRT1* can stimulate *AMPK* via the modulation of upstream *AMPK* kinase, liver kinase B1 [22]. Recent studies demonstrated that *AMPK/SIRT1* activation could protect ethanol-promoted liver diseases [22,23,24]. Our previous study also demonstrated that Gomisin N activates *AMPK* in ethanol-induced fatty liver in vivo and in vitro [25]. Therefore, the *AMPK* pathway and its downstream target genes have gained attention as potential targets for liver protection.

There is ample evidence suggesting that oxidative stress and lipid peroxidation also play a crucial role in the pathogenesis of ALD [12,26,27,28]. Alcohol exposure increases the activity of cytochrome P450 2E1 (*CYP2E1*), a major contributor to reactive oxygen species (ROS) generation, which is also involved in the development of fatty liver [29,30,31]. The inhibition of *CYP2E1* activity has been shown to result in successful recovery of ethanol-induced fatty liver [32]. Nuclear factor E2-related factor 2 (*Nrf2*) is another important regulator of the intracellular adaptive antioxidant response to oxidative stress [33,34,35]. In addition, alcohol exposure is shown to induce hepatic lipid accumulation in *Nrf2*-null mice [36], and the activation of *Nrf2* was able to successfully prevent alcohol-induced fatty liver [37]. Moreover, *SIRT1* can regulate transcription factors, such as *Nrf2* and *NF-κB*, which are involved in the regulation of antioxidant genes in the face of oxidative damage and suppression of pro-inflammatory cytokines, respectively [38,39,40,41,42]. Therefore, the amelioration of oxidative stress by the activation of antioxidant genes and regulation of inflammatory genes could be another effective mode of action against ethanol-induced liver injury.

Based on the evidence from above studies, we investigated whether CT has a protective effect against alcohol-induced hepatotoxicity while using a chronic-ethanol-drinking model in mice in vivo and in vitro using HepG2 and AML-12 cells. We examined the effects of CT on the de novo lipogenesis, fatty acid oxidation, oxidative stress and inflammation, and measured *AMPK/SIRT1* signaling to elucidate the underlying mechanism of the protective effect of CT against ethanol-induced liver injury. To our knowledge, this is first study elucidating the mechanism underlying the hepatoprotective effects of CT against ethanol-induced fatty liver.

## 2. Results

### 2.1. CT Countered Ethanol-Promoted Hepatic Steatosis in Chronic Ethanol-Fed Mice

**Figure 1:**
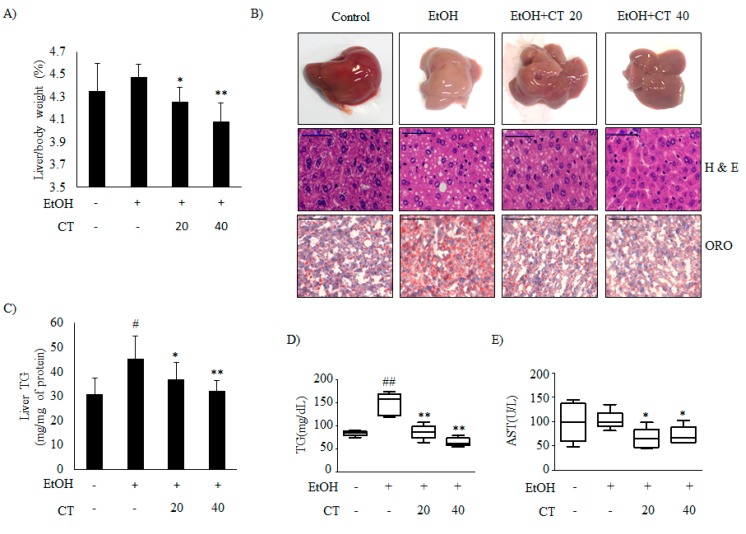
Cryptotanshinone (CT) countered ethanol-promoted hepatic steatosis in chronic ethanol-fed mice. C57BL/6 mice were given feeding control or ethanol-containing diet with or without CT (20 or 40 mg/kg) for four weeks. (**A**) Liver body index (%). (**B**) Liver morphology (upper), hematoxylin and eosin (H&E) staining (middle), and Oil Red O (ORO) staining (bottom) (scale bar = 50 μm). The images shown are representative of H&E and ORO staining. NAFLD activity score (NAS) for H&E staining is given in Supplementary Figure S1B. (**C**) Triglyceride (TG) levels of liver respective groups. (**D**) Serum levels of TG. (**E**) Serum levels of *AST*. Data are shown as mean ± SD (n = 6). ^#^ *p* < 0.05, ^##^ *p* < 0.01 vs. pair-fed control mice, * *p* < 0.05, ** *p* < 0.01 vs. ethanol-fed mice.

It is well known that ethanol exposure induces hepatic steatosis [12]. We established a chronic alcohol feeding mouse model while using C57BL/6 mice to evaluate the effects of CT on ethanol-promoted hepatic steatosis. Hence, the mice were randomly divided into the four groups (n = 10/group): control, ethanol, ethanol + CT 20 mg/kg, and ethanol + CT 40 mg/kg. During the experiments for four weeks, ethanol feeding led to body weight loss. CT administration did not affect body weight changes in ethanol feeding mice group (Supplementary Figure S1A). After four weeks of treatment, the liver index in ethanol-fed group (4.477 ± 0.113%) was higher than that in the control group (4.353 ± 0.241%), and it was significantly decreased by CT treatment at both low dose (20 mg/kg) (4.255 ± 0.129%) and high dose (40 mg/kg) (4.085 ± 0.164%) (Figure 1A). In addition, white-colored fatty livers were observed in ethanol-fed mice, whereas the CT-treated groups had healthy livers (Figure 1B, upper). Liver sections of the chronic ethanol exposure group that were stained with with haematoxylin and eosin (H&E) showed fat deposits, and Oil Red O (ORO) staining revealed the accumulation of lipid droplets (Figure 1B, middle and bottom). The NAFLD activity score (NAS) for H&E staining is given in Supplementary Figure S1B. However, CT treatment was able to significantly decrease the ethanol-induced hepatic fat deposition. In addition, ethanol-induced hepatic TG accumulation was significantly suppressed by CT administration (Figure 1C), consistent with the results of H&E and ORO staining. Serum biochemistry showed that the TG levels were elevated in the chronic ethanol-fed group, and the elevated TG levels were effectively decreased by CT treatment (Figure 1D). The aspartate aminotransferase (AST) levels were not only induced by ethanol consumption, but CT administration significantly reduced the *AST* levels as compared to only ethanol-treated group (Figure 1E). Meanwhile, alanine aminotransferase (ALT) levels were also not induced in only the ethanol-fed group, but CT administration tended to decrease ALT levels when compared to only ethanol-treated group (Supplementary Figure S1C). Also, ADH1 mRNA increased in mice treated with ethanol only and in mice treated with ethanol plus CT (Supplementary Figure S1D); however, ALDH2 expression also significantly increased in the CT treatment groups (Supplementary Figure S1E). These findings suggest that oxidation of ethanol to acetaldehyde and ALDH2 overexpression may detoxify acetaldehyde in the liver of CT-treated mice. Altogether, ethanol promoted hepatic steatosis with liver injury, which was ameliorated by CT treatment in chronic ethanol-fed mice.

### 2.2. CT Mitigates Ethanol-Induced TG Accumulation by Regulating Lipogenesis and Fatty Acid Oxidation in Chronic Ethanol-Fed Mice and HepG2 Cells

**Figure 2:**
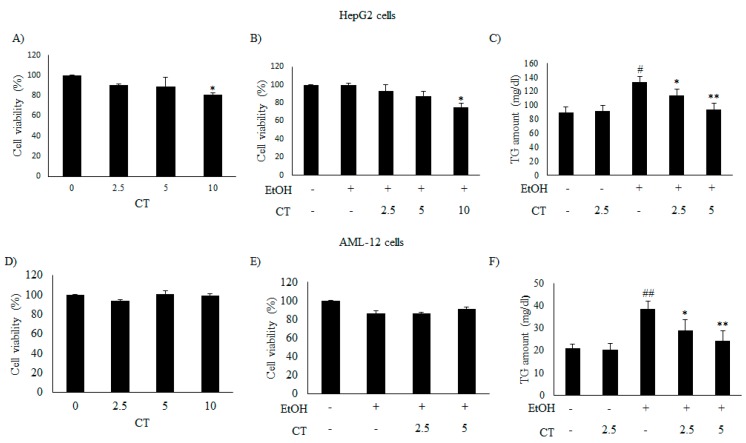
CT attenuated ethanol-induced intracellular TG accumulation in HepG2 and AML-12 cells. (**A**,**B**,**D**,**E**) HepG2 and AML-12 cells were treated with only CT at indicated concentrations and combined with 50 mM ethanol for 24 h. Cell viability was assessed by 3-(4,5- dimethylthiazol-2-yl)-2,5-diphenyltetrazolium bromide (MTT) assay. (**C**,**F**) HepG2 and AML-12 cells were treated with 50 mM ethanol in the presence or absence of CT (2.5 or 5 μM) for 24 h. Measurement of intracellular TG levels in HepG2 and AML-12 cells. Values are mean ± SD from triplicate experiments. ^#^ *p* < 0.05, ^##^ *p* < 0.01 vs. untreated control, * *p* < 0.05, ** *p* < 0.01 vs. ethanol-treated group.

We evaluated the inhibitory effects of CT on intracellular TG accumulation in ethanol-treated HepG2 and AML12 cells to confirm the in vivo protective effect of CT against hepatic steatosis. First, we examined the cytotoxicity of only CT or ethanol, and that of a combination of CT and ethanol to HepG2 and AML-12 cells. Both cell types were treated with 50 mM ethanol in the presence or absence of CT (2.5, 5.0, or 10 μM) for 24 h and 3-(4,5- dimethylthiazol-2-yl)-2,5-diphenyltetrazolium bromide (MTT) assay was performed. CT treatment produced no inhibitory effects on the viability of both cell types (Figure 2A,D) and a combination of 2.5 or 5.0 μM CT and 50 mM ethanol exhibited no cytotoxicity in both cell types (Figure 2B,E). Therefore, the co-treatment of both cell types with CT and ethanol was performed while using 2.5 or 5.0 μM CT and 50 mM ethanol in subsequent experiments. Subsequently, we assessed the potential inhibitory effects of CT on intracellular TG accumulation in ethanol-treated cells. As shown in Figure 2C,F, CT treatment significantly inhibited the ethanol-induced intracellular TG accumulation in both cell types. These findings suggest that CT can inhibit ethanol-stimulated intracellular TG accumulation in HepG2 and AML-12 cells. 

**Figure 3:**
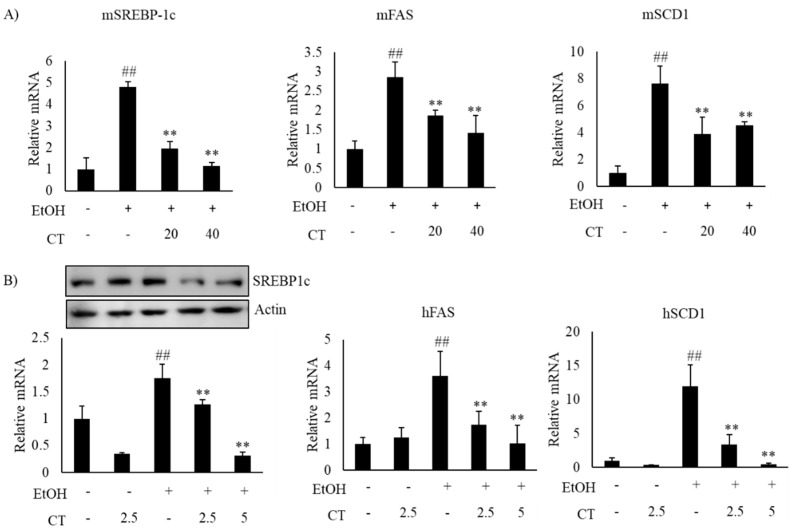
CT decreased the lipogenesis genes in ethanol-fed mice and HepG2 cells. (**A**) C57BL/6 mice were pair-fed either control or ethanol-containing diet with or without CT (20 or 40 mg/kg) for 4 weeks. qPCR analysis of m*SREBP-1c*, m*FAS*, and m*SCD1*. Data are shown mean ± SD (*n* = 6). ^#^ *p* < 0.05, ^##^ *p* < 0.01 vs. pair-fed control mice, * *p* < 0.05, ** *p* < 0.01 vs. ethanol-fed mice. (**B**) HepG2 cells were incubated with 50 mM ethanol and treated with CT (2.5 or 5 μM) for 24 h. Western blot and qPCR analysis of h*SREBP-1* and qPCR analysis of h*FAS*, and h*SCD1*. Values are mean ± SD from triplicate experiments ^##^ *p* < 0.01 vs. untreated control, ** *p* < 0.01 vs. ethanol-treated group.

**Figure 4:**
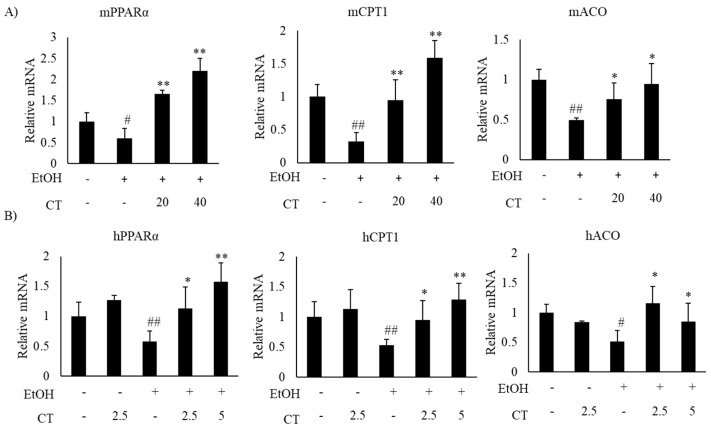
CT stimulated fatty acid oxidation in ethanol-fed mice and HepG2 cells. (**A**) C57BL/6 mice were pair-fed either control or ethanol-containing diet with or without CT (20 or 40 mg/kg) for 4 weeks. qPCR analysis of m*PPARα*, m*CPT1*, and m*ACO*. Data are shown mean ± SD (*n* = 6). ^#^ *p* < 0.05, ^##^ *p* < 0.01 vs. pair-fed control mice, * *p* < 0.05, ** *p* < 0.01 vs. ethanol-fed mice. (**B**) The HepG2 cells were incubated with 50 mM ethanol and treated with CT (2.5 or 5 μM) for 24 h. qPCR analysis of h*PPARα*, h*CPT1*, and h*ACO*. Values are mean ± SD from triplicate experiments. ^#^ *p* < 0.05, ^##^ *p* < 0.01 vs. untreated control, * *p* < 0.05, ** *p* < 0.01 vs. ethanol-treated group.

Alcohol exposure enhances lipogenesis and impairs fatty acid oxidation, which leads to the development of hepatic steatosis [12,43]. Hence, we investigated the expression of lipogenesis- and fatty acid oxidation-related genes in the liver of ethanol-fed mice and HepG2 cells, with or without CT treatment. The expression of sterol regulatory element-binding protein-1c (*SREBP-1c*) at mRNA and protein levels, which is a key transcription factor that regulates lipogenesis and its downstream lipogenesis genes, including fatty acid synthase (*FAS*) and stearoyl-CoA desaturase-1 (*SCD1*), were increased in ethanol-fed mice and ethanol-treated HepG2 cells, but CT treatment was able to reduce the expression of lipogenesis-related genes (Figure 3A,B). Moreover, the expressions fatty acid oxidation genes, such as peroxisome proliferator-activated receptor α (*PPARα*), carnitine palmitoyltransferase-1α (*CPT1*), and acyl-coenzyme A oxidase (*ACO*), were significantly reduced in ethanol-fed mice and HepG2 cells, and CT administration significantly reversed these ethanol-induced effects (Figure 4A,B). These results suggest that CT could ameliorate ethanol-induced steatosis by decreasing lipogenesis and increasing fatty acid oxidation in chronic ethanol-fed mice and HepG2 cells.

### 2.3. CT Activated AMPK/SIRT1 Signaling in Ethanol-Treated Mice and HepG2 Cells

**Figure 5:**
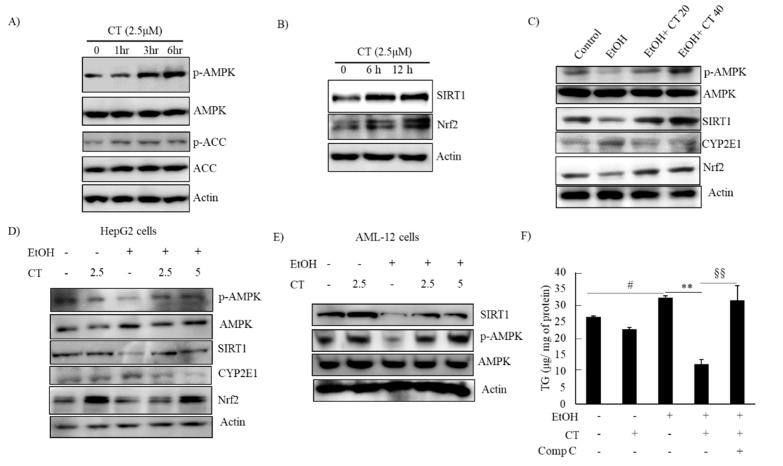
CT activated *AMPK/SIRT1* signaling. (**A**,**B**) HepG2 cells were treated with 2.5 μM CT for indicated times. Western blot analysis of phosphorylated *AMPK*, *ACC*, *SIRT1*, and *Nrf2*. (**C**) C57BL/6 mice were pair-fed either control or ethanol-containing diet with or without CT (20 or 40 mg/kg) for four weeks. Western blot analysis of phosphorylated *AMPK*, *SIRT1. CYP2E1*, and *Nrf2*. (**D**) HepG2 cells were incubated with 50 mM ethanol and treated with CT (2.5 or 5 μM) for 24 h. Western blot analysis of phosphorylated *AMPK*, *SIRT1*, *CYP2E1*, and *Nrf2*. (**E**) AML-12 cells were incubated with 50 mM ethanol and treated with CT (2.5 or 5 μM) for 24 h. Western blot analysis of phosphorylated *AMPK* and *SIRT1*. The images are representative (**F**) HepG2 cells were pretreated with CT (2.5 μM) for 3 h or with compound C (comp C) (10 μM) for 6 h, followed by ethanol (100 μM) treatment. Measurement of intracellular TG levels. Data are shown as mean ± SD of three independent experiments. ^#^ *p* < 0.05 vs. untreated control, ** *p* < 0.01 vs. ethanol-treated group. ^§^^§^ *p* < 0.01 vs. ethanol and CT-treated group. Densitometric analysis of western blots are given in Supplementary Figures S2 and S3A–G.

*AMPK/SIRT1* activation is a potential therapeutic target against ALD [22,23,24]. We evaluated the levels of phosphorylated *AMPK* and *SIRT1* protein in CT-treated HepG2 and AML-12 cells to decipher the underlying mechanisms involved in the protective effects of CT against ethanol-induced ALD. The levels of phosphorylated *AMPK* and *ACC*, a target of *AMPK* downstream, were increased by CT treatment after 3 h treatment, and the *SIRT1* protein was also increased by CT treatment after 6 h and 12 h treatment in HepG2 cells (Figure 5A,B). Subsequently, we further assessed the levels of phosphorylated *AMPK* and *SIRT1* in the liver of ethanol-fed mice and, HepG2 and AML-12 cells. Ethanol exposure exhibited the reduced levels of phosphorylated *AMPK*, and *SIRT1* protein in the liver of ethanol-fed mice (Figure 5C), and HepG2 (Figure 5D) and AML-12 cells (Figure 5E) as compared with control groups; however, CT administration efficiently restored them. HepG2 cells were pretreated with CT and/or compound C (*AMPK* Inhibitor) and Ex52735 (*SIRT1* inhibitor) before treatment with ethanol and TG accumulation was evaluated to further examine the role of *AMPK/SIRT1* in CT-mediated protection against ethanol-induced hepatic steatosis. As shown in Figure 5F, CT treatment significantly suppressed the ethanol-stimulated intracellular TG accumulation in HepG2 cells. However, co-treatment with compound C significantly blocked the CT-mediated reduction in TG accumulation, but not Ex52735 (Supplementary Figure S3H), which suggested that *AMPK* activation mediates CT countering ethanol-promoted hepatic steatosis. In addition, the stimulatory effects of CT on *AMPK/SIRT1* were also proven in AML-12 cells. As shown in Figure 5F, CT increased the levels of p-*AMPK* and SIRT1, and recovered ethanol-mediated reduction in those levels (Figure 5F). Collectively, these results demonstrated that CT stimulated hepatic *AMPK/SIRT1* signaling, which might act as defense mechanism of CT against ALD.

### 2.4. CT Prevents Ethanol-Induced Oxidative Stress in Chronic Ethanol-Fed Mice and HepG2 Cells

**Figure 6:**
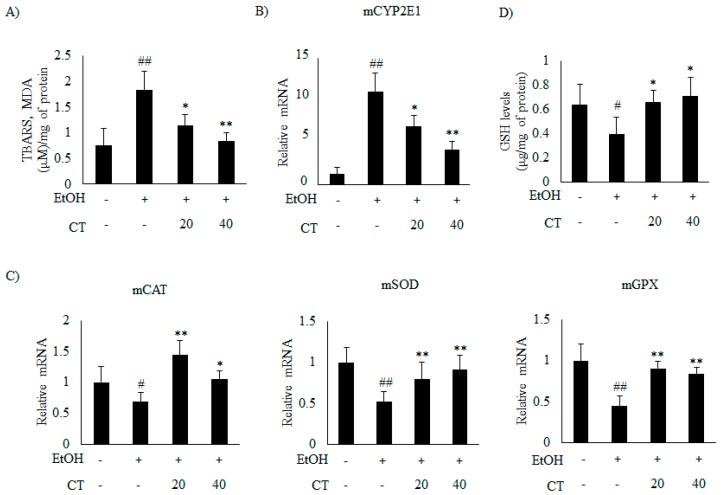
CT attenuated ethanol-induced oxidative stress in chronic ethanol-fed mice. C57BL/6 mice were pair-fed either control or ethanol-containing diet with or without CT (20 or 40 mg/kg) for four weeks. (**A**) *TBARS* levels of respective groups. (**B**) qPCR analysis of *CYP2E1*. (**C**) qPCR analysis of m*CAT*, m*SOD*, and m*GPX*. (**D**) Hepatic levels of glutathione (GSH). Data are expressed as mean ± SD (*n* = 6). ^#^ *p* < 0.05, ^##^ *p* < 0.01 vs. pair-fed control mice, * *p* < 0.05, ** *p* < 0.01 vs. ethanol-fed mice.

Previous studies suggest that oxidative stress and inflammation also play a crucial role in the pathogenesis of ALD [12]. Hence, we examined the effects of CT on oxidative stress and inflammation genes in the liver of ethanol-fed mice and HepG2 cells. First, we determined the hepatic TBARS levels (measured as malondialdehyde MDA, an indicator of lipid peroxidation) to evaluate the oxidative damage. The results showed that hepatic TBARS levels were significantly increased in the ethanol-fed mice, but CT administration significantly reduced the TBARS levels (Figure 6A). This result suggested that CT protected the mice against ethanol-induced oxidative stress. 

It has been reported that *CYP2E1* plays a critical role in ethanol-induced ROS generation [31]. Thus, we examined whether *CYP2E1* inhibition and improvement of the antioxidant defense system mediated the protective effect of CT against oxidative stress. The results revealed that the protein and the mRNA levels of *CYP2E1* increased upon ethanol consumption in ethanol fed mice. 

**Figure 7:**
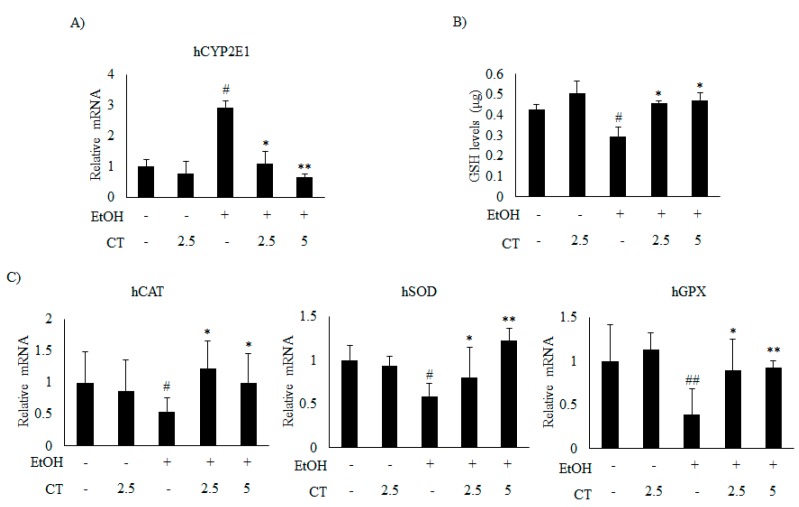
CT inhibited ethanol-induced oxidative stress in HepG2 cells. HepG2 cells were incubated with 50 mM ethanol and treated with CT (2.5 or 5 μM) for 24 h. (**A**) qPCR analysis of *CYP2E1*. (**B**) Measurement of intracellular GSH levels. (**C**) qPCR analysis of h*CAT*, h*SOD*, and h*GPX*. Data are shown as mean ± SD from triplicate experiments. ^#^ *p* < 0.05, ^##^ *p* < 0.01 vs. untreated control, * *p* < 0.05, ** *p* < 0.01 vs. ethanol-treated group.

Which was significantly reduced by CT treatment (Figure 5C and Figure 6B). Moreover, ethanol exposure significantly reduced the antioxidant genes, such as catalase (CAT), superoxide dismutase (SOD), and glutathione peroxidase (GPX) (Figure 6C) and hepatic glutathione (GSH) levels (Figure 6D), while CT treatment significantly reversed these ethanol-mediated effects. The in vivo inhibitory effects of CT on oxidative stress was also examined in HepG2 cells. Ethanol treatment increased the protein and mRNA levels of *CYP2E1* in HepG2 cells (Figure 5D and Figure 7A); however, CT prevented the increase. Furthermore, ethanol treatment reduced both GSH levels (Figure 7B) and the antioxidant genes including CAT, SOD, and GPX at mRNA levels (Figure 7C), but CT treatment prevented the ethanol-mediated reduction in HepG2 cells. Taken together, our results suggest that CT prevents ethanol-induced oxidative stress in ethanol-fed mice and HepG2 cells.

Moreover, *AMPK/SIRT1* can regulate transcription factor *Nrf2*, which is an important modulator of the antioxidant defense response to oxidative stress by upregulating antioxidant genes expression [39,40,42]. Thus, we investigated whether *Nrf2* is also involved in the protective effects of CT against oxidative stress. CT treatment increased the *Nrf2* levels in a time dependent manner in HepG2 cells (Figure 5B). Afterwards, we further assessed the expression of *Nrf2* in ethanol-fed mice and HepG2 or AML-12 cells. The *Nrf2* protein levels were markedly decreased in both ethanol-fed mice (Figure 5C) and ethanol-treated HepG2 (Figure 5D) or AML12 cells (Figure 5E) as compared to the control groups, but CT treatment significantly reversed these effects. These findings suggest that the upregulation of *Nrf2* may be also involved in CT-mediated safeguard against ethanol-induced oxidative stress. Furthermore, to investigate whether *AMPK* plays a role in CT-induced *Nrf2* expression, we examined *Nrf2* expression in CT-incubated HepG2 cells that were pretreated with compound C. Pretreatment with compound C efficiently blocked the CT-induced increase in the *Nrf2* protein level (Supplementary Figure S4), which suggested that *AMPK* activation is involved in the CT-induced activation of *Nrf2*.

### 2.5. CT Prevents Ethanol-Induced Inflammation in Chronic Ethanol-Fed Mice and HepG2 Cells

**Figure 8:**
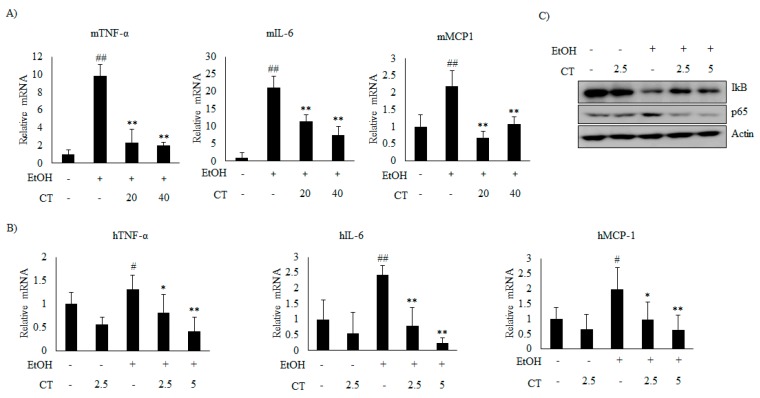
CT inhibited inflammation induced by ethanol in chronic ethanol-fed mice and HepG2 cells. (**A**) qPCR analysis of m*TNF-α*, m*IL-6*, and m*MCP-1*. Data are shown as mean ± SD (n = 6). ^##^ *p* < 0.01 vs. pair-fed control mice, * *p* < 0.05, ** *p* < 0.01 vs. ethanol-fed mice. (**B**) qPCR analysis of h*TNF-α*, h*IL-6*, and h*MCP-1*. Data are expressed as mean ± SD from three independent experiments. ^#^ *p* < 0.05, ^##^ *p* < 0.01 vs. untreated control, * *p* < 0.05, ** *p* < 0.01 vs. ethanol-treated group. (**C**) HepG2 cells were incubated with 50 mM ethanol and treated with CT (2.5 or 5 μM) for 24 h. Western blot analysis of *IκB* and *NF-κB p65*. Densitometric analysis of western blots is given in Supplementary Figure S5.

Next, we analyzed the effect of CT on inflammation in ethanol-fed mice and HepG2 cells. Ethanol exposure increased the mRNA levels of inflammatory genes, including tumor necrosis factor α (TNFα), interleukin 6 (IL-6), and monocyte chemoattractant protein 1 (MCP1), but CT significantly inhibited the ethanol-induced those inflammatory genes, which suggested that CT exerts anti-inflammatory effects in ethanol-fed mice and HepG2 cells, as shown in Figure 8A,B. Further, ethanol exposure reduced the level of *NF-κB* inhibitory protein (IκB), while increasing the level of p65, a subunit of *NF-κB* (Figure 8C). However, CT treatment reversed both of these effects. These results indicate that CT inhibits ethanol-induced inflammation in HepG2 cells. These findings suggest that CT ameliorates ethanol-stimulated inflammation in ethanol-fed mice and HepG2 cells.

## 3. Discussion

There are currently no approved therapies to treat non-alcoholic fatty liver disease, as well as alcoholic fatty liver disease. Herbal medicines have garnered significant attention in recent times, as they have very few side effects. Hence, we examined the protective effects of CT against chronic ethanol-induced fatty liver in vivo while using mice as well as in vitro using HepG2 cells. The results revealed that CT attenuates ethanol-induced hepatic injury via the inhibition of lipogenesis-related genes, oxidative stress, and inflammation. 

In this present study, we first investigated whether CT attenuated ethanol-induced liver injuries in ethanol fed mice. The results revealed that the TG levels were elevated in the serum of chronic ethanol-fed mice, and the elevated TG levels were effectively decreased by CT treatment. Unexpectedly, the levels of *AST* and ALT were not significantly induced by only ethanol consumption, but CT administration decreased the levels compared to only ethanol-treated control group. Following oral ingestion, ethanol is oxidized to acetaldehyde by alcohol dehydrogenase (ADH) prior to further oxidization into acetate by aldehyde dehydrogenase (ALDH) [44]. Acetaldehyde, which is a major by-product of ethanol, is one of the predominant compounds that are highly toxic to hepatocytes. ALDH2 is a key enzyme for acetaldehyde detoxification in the liver [45]. Moreover, ALDH2 deficiency is associated with increased acetaldehyde and glucocorticoids after alcohol consumption [45]. We measured ADH1 and ALDH2 expression in mouse livers while using qPCR. The results revealed that ADH1 mRNA increased in mice treated with ethanol only and in mice treated with ethanol plus CT (Supplementary Figure S1D). However, ALDH2 expression also significantly increased in the CT treatment groups (Supplementary Figure S1E). These findings suggest that oxidation of ethanol to acetaldehyde and ALDH2 overexpression may detoxify acetaldehyde in the liver of CT-treated mice. In addition, hepatic TG levels were significantly elevated in the liver of chronic ethanol-fed mice and these elevated TG levels were effectively decreased by CT treatment. These findings indicated that CT prevents ethanol-induced lipid accumulation in the liver of ethanol fed mice. 

As mentioned earlier, there are multiple mechanisms that are associated with the development of alcoholic fatty liver [12,15]. Alcohol exposure is known to enhance lipogenesis by the upregulation of *SREBP-1c* and its target lipogenesis-related genes, including *FAS*, *SCD1*, and ACC, which leads to hepatic steatosis [46,47]. Meanwhile, alcohol-induced impairment of fatty acid oxidation via the inhibition of *PPARα*, which controls the expression of *CPT1* and *ACO*, also plays a pivotal role in the progresssion of hepatic steatosis [12,43]. Evidence suggests that the activation of *AMPK* increases fatty acid oxidation and decreases lipogenesis [15,18,19,20]. As previously mentioned, *AMPK/SIRT1* activation is a potential therapeutic target against ALD [22,23,24]. Hence, we evaluated the levels of phosphorylated *AMPK* and *SIRT1* protein in CT-treated HepG2 cells. Of note, CT treatment significantly increased the phosphorylated form of *AMPK* and ACC, a target of *AMPK* downstream, and the *SIRT1* protein in a time dependent manner in HepG2 cells. It has been reported that the activation of *AMPK* plays an essential role in the protection of hepatic steatosis by suppressing ACC activity via phosphorylation for the inhibitory effect of fatty acid synthesis, and inhibiting both the transcriptional activity and the expression of *SREBP-1c* and its target lipogenesis genes. In the present study, we observed that CT efficiently increased the phosphorylated ACC and down-regulated the expression of *SREBP-1c* and its target lipogenesis genes including *FAS*, SCD1, and ACC in ethanol-fed mice and ethanol-treated HepG2 cells. In addition, CT treatment significantly inhibited hepatic TG accumulation, which was consistent with the suppression of lipogenesis-related genes and the increased expression of fatty acid oxidation-related genes, such as *PPARα*, *CPT1*, and *ACO*. Additionally, CT-treatment restored ethanol-reduced the levels of phosphorylated *AMPK* and *SIRT1* protein in the liver of ethanol-fed mice and HepG2 cells. Interestingly, compound C (*AMPK* Inhibitor) significantly inhibited ethanol-induced intracellular TG accumulation in HepG2 cells. However, co-treatment with compound C significantly blocked the CT-mediated reduction in TG accumulation, but not Ex52735 (*SIRT1* inhibitor), which suggested that *AMPK*, as an upstream of SIRT1, mediates CT-induced protection against ethanol-promoted hepatic steatosis. Based on these results, CT-mediated-*AMPK/SIRT1* activation might play a critical role in the protective effects of CT against alcoholic fatty liver.

*CYP2E1* is a major culprit to ROS-generation-mediated fatty liver formation [29,30,31]. The elevation of lipid peroxidation has been reported in both acute and chronic ethanol-fed mouse models [48,49]. In addition, GSH is an important intracellular antioxidant enzyme that plays a pivotal role in antioxidant defense and detoxification [50]. Ethanol is also involved in the disruption of antioxidant activity, via the depletion of GSH levels and creation of favorable conditions for oxidative stress [51]. In the present study, ethanol increased the expression of *CYP2E1* and TBARS, while reducing the hepatic GSH levels as well as antioxidant genes, such as CAT, SOD, and GPX. However, CT treatment significantly inhibited TBARS, and it led to a recovery of GSH levels and the antioxidant genes, suggesting that CT has antioxidant activity, which might be involved in the prevention of ALD. 

Moreover, *AMPK* and *SIRT1* can regulate transcription factors, such as *Nrf2* and *NF-κB*, which are involved in regulating antioxidant genes against oxidative damage and the suppression of pro-inflammatory cytokines, respectively [39,40,42]. *Nrf2*, which is an essential transcription factor for expression of antioxidant genes, is another important modulator of the intracellular adaptive antioxidant response to oxidative stress [33,34]. *Nrf2* binds to specific DNA sequence antioxidant responsive element (ARE) and stimulates the transcription of downstream target genes, antioxidant genes, including CAT, SOD, and GPX. In the current study, *Nrf2* proteins were significantly decreased in ethanol-fed mice and ethanol-treated HepG2 cells, but these effects were significantly reversed in the CT-treated groups, which is consistent with upregulation of antioxidants genes, such including CAT, SOD, and GPX, suggesting that *Nrf2* might play a role in CT-mediated antioxidant activity. Importantly, the inhibition of *AMPK* using compound C prevented the CT-induced increase in *Nrf2* protein, indicating that *AMPK* activation is involved in CT-induced activation of *Nrf2*. Taken together, these results suggest that *AMPK/SIRT1* and *Nrf2* signaling may be also involved in the protective effects of CT against ethanol-mediated oxidative stress. 

The increased expression of hepatic cytokines mediates the progression of alcoholic hepatic steatosis in ethanol-fed mice model [52]. Previous studies suggest that cytokines, such as interleukins and TNF-α, play important roles in acute and chronic inflammation [12]. The key transcription factor, *NF-κB*, stimulates inflammatory genes, such as TNF-α, IL-6, and MCP-1. In our study, CT reversed the ethanol-induced increase in the *NF-κB p65* protein level, and restored the IκB level. Consistent with the above results, CT significantly inhibited the ethanol-induced inflammatory genes, such as TNF-α, IL-6, and MCP-1 at mRNA levels. Similar to these findings, cryptotanshinone demonstrated anti-inflammatory properties by inhibiting the expression of NO, iNOS, and COX-2 in RAW 264.7 cells [8]. These results revealed that CT has anti-inflammatory properties, which might help to prevent the progression of alcoholic hepatic steatosis.

In addition, previous study demonstrated that *AMPK/SIRT* activation inhibits the NF-kB-mediated inflammation pathway, which leads to repressing the expression of inflammatory cytokine genes [53]. In the current study, CT significantly blocked the ethanol-induced increase in *NF-κB p65* level by inducing the phosphorylation of IκB, and inhibited the expression of inflammatory cytokine gens, including TNF-α, IL-6, and MCP-1, which suggested that *AMPK/SIRT1* pathway might be also involved in the protective effects of CT against ethanol-mediated inflammation.

*S. miltiorrhiza* is popularly used for treating liver diseases, including hepatitis and cirrhosis, in many Asian countries. The previous study demonstrated that a standardized fraction from root of *S. miltiorrhiza* (10 μg/mL) and CT (10 μM) suppressed alcohol-induced lipid accumulation and inhibited *SREBP-1* in primary rat hepatocytes [10]. Moreover, tanshinone IIA (10 μM) protected from lipopolysaccharides (LPS)—and ethanol-induced hepatotoxicity in RAW 264.7 cells [54]. It has also been showed that the purified extract of *S. miltiorrhiza* (PF2401-SF) protected against liver injury at 25–100 mg/kg in rats, which was more potent than the ethanol extract of *S. miltiorrhiza.* In our present study, a lower concentration of CT inhibited alcohol-induced steatosis, oxidative stress and inflammation in ethanol-fed mice (20 and 40 mg/kg) and HepG2 cells (5 μM). These findings suggest that CT is the major active component of *S. miltiorrhiza* inhibiting alcohol-induced liver injury. 

## 4. Conclusions

**Figure 9:**
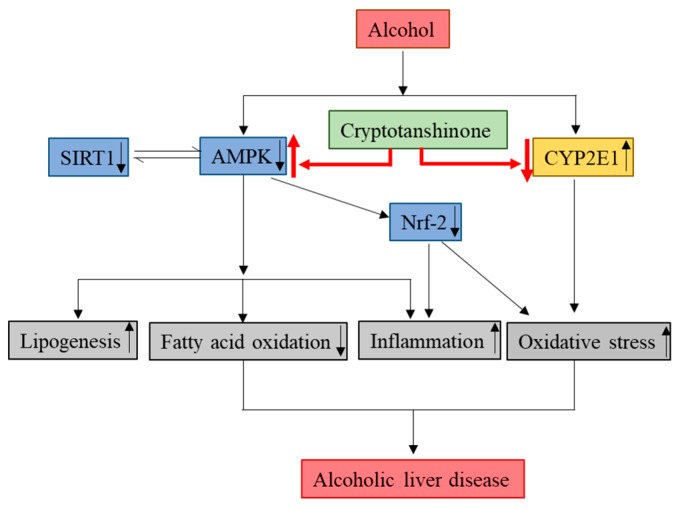
Molecular mechanisms involved in the protective effects of CT against ALD. Cryptotanshinone (CT) attenuates ethanol-induced hepatic steatosis, oxidative stress, and inflammation. The activation of *AMPK/SIRT1* and inhibition of *CYP2E1* might be involved in hepatoprotective effects of CT against ethanol-induced liver. ↑ means upregulation and ↓ means downregulation.

In the present study, we have demonstrated that CT attenuates ethanol-induced hepatic steatosis via the inhibition of lipogenesis genes, oxidative stress, and inflammation for the first time in chronic ethanol-fed mice and HepG2 cells. The activation of *AMPK/SIRT1*, *Nrf2*, and inhibition of *CYP2E1* might be involved in the protective effects of CT against ALD (Figure 9). The findings of this study suggest that CT could be an effective therapeutic agent for the treatment of ethanol-induced liver injury. 

## 5. Materials and Methods

### 5.1. Reagents

Cryptotanshinone (≥98% purity) was obtained from ChemFaces (Wuhan, China). Antibodies against *SREBP-1c* (H-160; sc-8984), *PPARα* (H-2; sc-398394), IkBα (H-4; sc-164), NFκB p65 (F-6; sc-8008p65), *AMPK* α1/2 (D-6; sc-74461), *SIRT1* (H-300; sc-15404), and β-actin (C4; sc-47778) were purchased from Santa Cruz Biotechnology (Santa Cruz, CA, USA). Specific primary antibodies against p-ACC (Ser79; #3661s) and ACC (C83B10; #3676s) were purchased from Cell Signaling Technology (Danvers, MA, USA). Antibodies against CYP2E1 (ab28146) and pAMPK α1 (Thr172; PA5-17831) were purchased from Abcam (Cambridge, MA, USA) and Invitrogen (Rockford, IL, USA), respectively. The *Nrf2* antibody (NBP1-32822) was obtained from Novus Biologicals (Centennial, CO, USA). Ethanol, Ex52735, and compound C were obtained from Sigma (St. Louis, MO, USA). 

### 5.2. Cell Culture

The human hepatocarcinoma cell line, HepG2, was obtained from American Type Culture Collection (Manassas, VA, USA) and grown in DMEM that was supplemented with 10% heat-inactivated FBS, penicillin (100 units/mL), and streptomycin sulfate (100 μg/mL). The murine cell line, AML-12 (non-cancerous), was obtained from Dr. SK Lee (Kyungpook National University, South Korea) and grown in DMEM/F12 medium supplemented with 10% heat-inactivated FBS, penicillin (100 units/mL), and streptomycin sulfate (100 μg/mL), 1× Insulin-Transferrin-Selenium (ITS-G) mixture, and 40 ng/mL dexamethasone. The cells were maintained at 37 °C in an atmosphere of 5% CO_2_. 

### 5.3. Cytotoxicity Assay

The HepG2 and AML-12 cells were incubated with CT for 3 h and then treated with 50 mM ethanol for 24 h. The inhibitory effects of CT and ethanol on HepG2 and AML-12 cells were assessed while using a 3-(4,5-dimethylthiazol-2-yl)-2,5-diphenyltetrazolium bromide (MTT) assay, as per the manufacturer’s instructions (Promega, Madison, WI, USA). 

### 5.4. Animal Experiments

Pusan National University’s Institutional Animal Care and Use Committee approved all of the animal experiments, which were in accordance with the established ethical and scientific care procedures (PNU-2018-1969). Male C57BL/six mice (eight-week-old, 20–22 g) were obtained from Doo Yeol Biotech (Seoul, South Korea). The mice were maintained at 22 ± 2 °C on a 12 h:12 h light-dark cycle and 50–60% relative humidity. For the experiments, the mice were randomly divided into the following four groups (n = 10/group): control, ethanol, ethanol + CT 20 mg/kg, and ethanol + CT 40 mg/kg. A model of chronic EtOH intake was used for feeding, according to a previously described NIAAA protocol [55]. The mice were given free access to the control diet or alcohol LiebereDeCarli liquid diet (Research Diets Inc., NJ, USA) for four weeks, and from third week onwards, CT (20 mg/kg or 40 mg/kg) was orally administered daily. After four weeks, the mice were euthanized, following which their blood and liver tissue were stored at −80 °C until further analyses.

### 5.5. TG Measurement

The TG levels of liver tissue lysates or HepG2 cells and AML-12 cells were measured, as described previously [56]. Briefly, TG were extracted while using chloroform/methanol (2:1), evaporated, and then dissolved in ethanol. TG content was determined while using an enzyme reaction kit (Asan Pharmaceutical, Seoul, Korea). The TG levels were normalized to the protein concentration.

### 5.6. Histopathological Analysis

Samples from the liver were separated, fixed in 10% buffered formalin and then embedded in paraffin, and sectioned (5 μm thickness) while using a frozen microtome (HM560H, Microm International, Walldorf, Germany). Subsequently, the sectioned tissues were stained with hematoxylin and eosin (H&E) and Oil Red O (ORO), following which, they were observed under a light microscope. The quantification of H&E staining based on steatosis, inflammation, and fibrosis was undertaken by NAFLD activity score (NAS) from the Nonalcoholic steatohepatitis-Clinical Research Network (NASH-CRN), as previously described [57].

### 5.7. Biochemical Analysis

ALT, AST, and TG levels of serum samples were determined while using FUJI DRI-CHEM 7000i (FUJI FILM, Tokyo, Japan). 

### 5.8. Quantitative Polymerase Chain Reaction (qPCR)

The total RNA was extracted from mice liver or HepG2 cells while using TRIzol^TM^ (Invitrogen, Carlsbad, CA, USA), according to the manufacturer’s instructions. The isolated RNA (1 µg) was converted in to cDNA using TOPScript RT DryMix (Enzynomics, Daejeon, Korea). Quantitative real-time PCR was analyzed using a SYBR Green premixed Taq reaction mixture with the gene-specific primers listed in Supplementary Table S1. The geometric mean of housekeeping gene 18S ribosomal RNA (18S rRNA) was used as an internal control to normalize the variability in expression levels. The expression levels were analyzed using the 2 -ΔΔCT method. as described previously [58].

### 5.9. Measurement of Hepatic Lipid Peroxidation and GSH Level

Hepatic lipid peroxidation was measured while using an OxiSelect™ TBARS assay kit-MDA Quantitation (Cell Biolabs Inc., San Diego, CA, USA). The GSH level in the liver was assessed using a kit (K261-100, BioVision, CA, USA). All of the measurements were carried out according to the manufacturers’ instructions.

### 5.10. Western Blot Analysis

The protein lysates were prepared from mice liver or HepG2 and AML-12 cells using Pro-Prep Protein Extraction Solution (Intron Biotechnology, Seoul, Korea), according to the manufacturer’s instructions. Equal amounts of protein (50 μg) were separated by 12% SDS-PAGE and then transferred to polyvinylidene difluoride membranes (Amersham Pharmacia Biotech, Amersham, UK). After transfer, the membranes were blocked in 5% nonfat skim milk, followed by incubation with primary antibodies (1:1000) overnight at 4 °C. After over night incubation, membranes were probed with anti-rabbit or anti-mouse secondary antibodies (1:1000) (Santa Cruz Biotechnology) conjugated to peroxidase, and the protein bands were detected by using an enhanced chemiluminescence system (ECL Advance, GE Healthcare, Hatfield, UK). 

### 5.11. Statistical Analysis

The data that are shown in this study are expressed as mean ± SD. The data were analyzed while using one-way ANOVA, and the differences between means were determined using the Tukey–Kramer post-hoc test. The sample size was calculated by assuming the effect size and the variance for NAS of pair-fed control and ethanol-fed mice based on literature data. The values were considered statistically significant at *p* < 0.05.

## Supplementary Materials

Supplementary materials can be found at https://www.mdpi.com/1422-0067/21/1/265/s1.

## Abbreviations

ACC	Acetyl-CoA carboxylase
ACO	Acyl-coenzyme A oxidase
ALD	Alcoholic liver disease
ALT	Alanine aminotransferase
AMPK	AMP-activated protein kinase
AST	Aspartate aminotransferase
*CYP2E1*	Cytochrome P450 2E1
CPT1α	Carnitine palmitoyltransferase-1α
CAT	Catalase
CT	Cryptotanshinone
EtoH	Ethanol
FAS	Fatty acid synthase
GPX	Glutathione peroxidase
GSH	Glutathione
H&E	Haematoxylin and eosin
IκB	I Kappa B-alpha
IL6	Interleukin 6
MCP-1	Monocyte chemotactic protein-1
NF-κB	Nuclear factor-kappa B
*NRF2*	Nuclear factor erythroid-derived 2-related factor 2
ORO	Oil Red O
PPAR α	Peroxisome proliferator-activated receptor α
qPCR	Quantitative polymerase chain reaction
ROS	Reactive oxygen species
SCD1	Stearoyl-CoA desaturase-1
SIRT1	Sirtuin 1
SOD	Superoxide dismutase
SREBP-1c	Sterol regulatory element-binding protein-1c
TBARS	Thiobarbituric acid reactive substances
TG	Triglyceride
TNFα	Tumor necrosis factor-α
