# Supplementary material for: Cryptotanshinone from the Salvia miltiorrhiza Bunge Attenuates Ethanol-Induced Liver Injury by Activation of AMPK/SIRT1 and Nrf2 Signaling Pathways
Source: Int J Mol Sci. 2019 Dec 30;21(1):265. doi: 10.3390/ijms21010265 (PMC6981483; doi:10.3390/ijms21010265)
Supplement: Supplementary file 1 [file ijms-21-00265-s001.pdf]

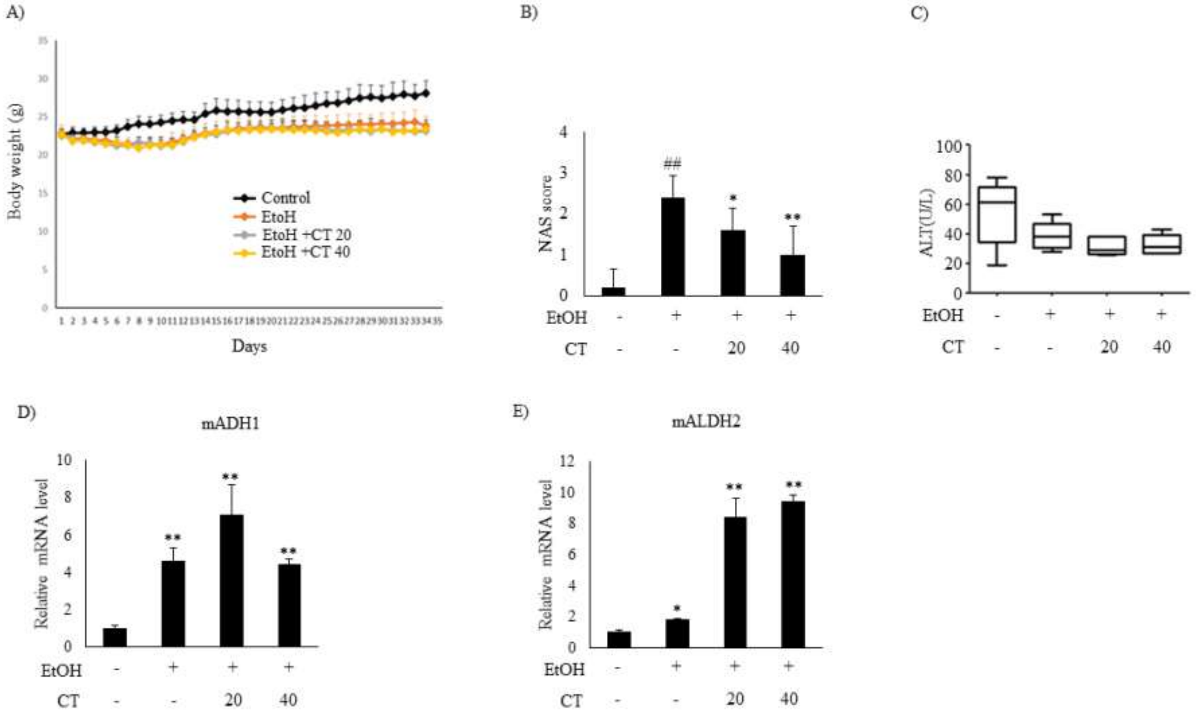

Figure 1. Cryptotanshinone (CT) attenuates ethanol-promoted hepatic steatosis in chronic ethanol-fed mice.

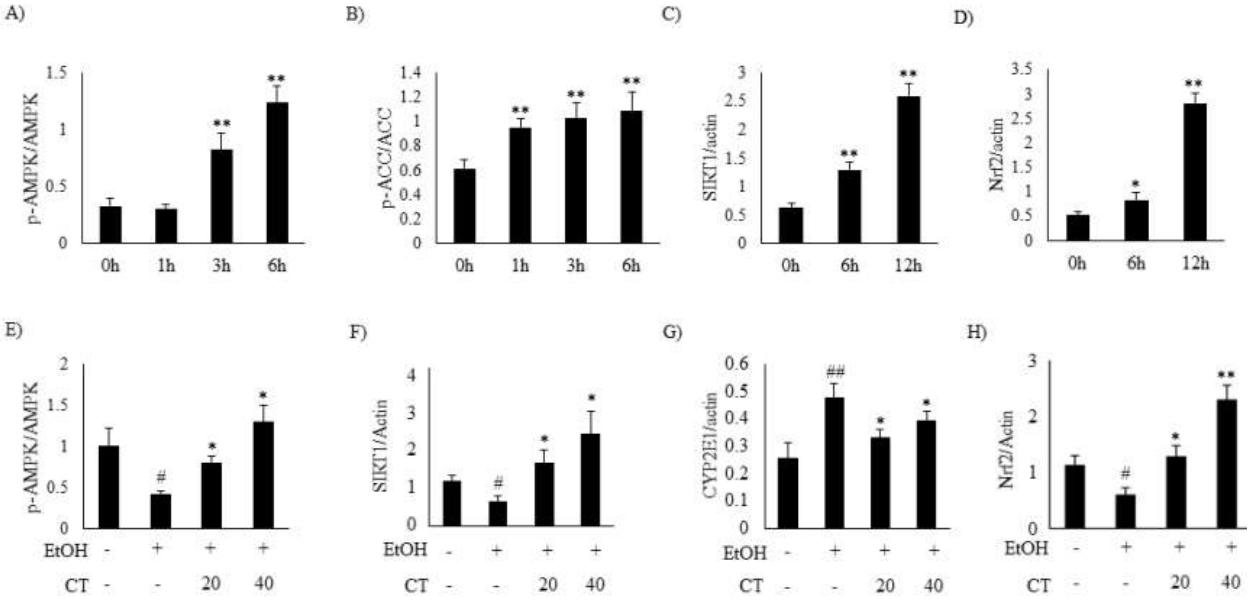

Figure 2. Densitometric analysis of western blots shown in figure 5A, 5B and 5C.

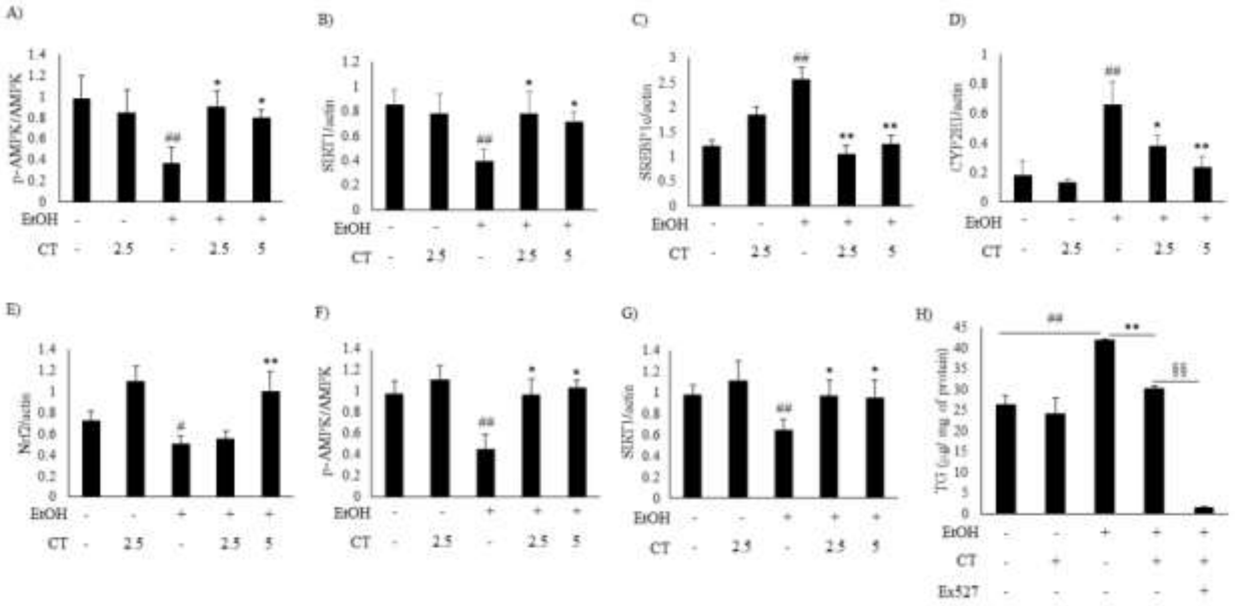

Figure 3. Densitometric analysis of western blots shown in figure 5D and 5D.

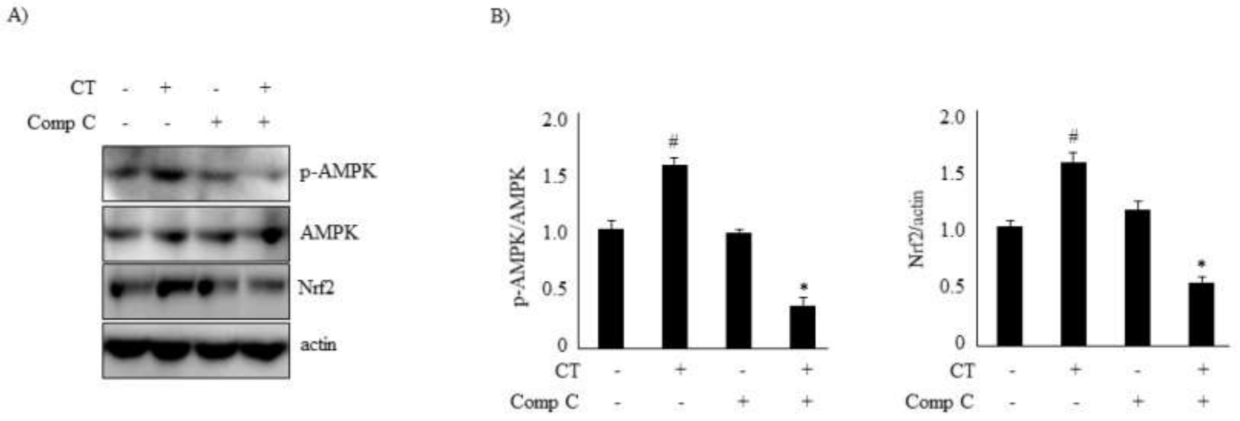

Figure 4. Compound C efficiently blocked the CT-induced *Nrf2* protein level in HepG2 cells.

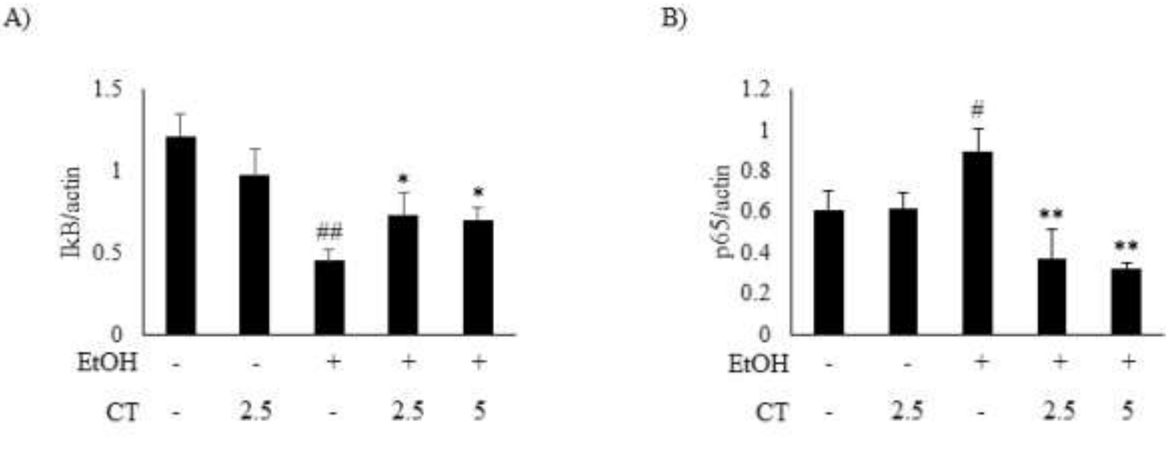

Figure 5. Densitometric analysis of western blots shown in figure 8C.

Supplementary Table S1. qPCR primers list.

Human

Gene	Forward primer	Reverse primer
hSREBP1c	CGGAGCCATGGATTGCACT	TAGGCCAGGGAAGTCACTG
hCYP2E1	AGCAGATTTCTGGGAGCCTCAGTT	AGTGGAACTGTGACTTGGAGCTGT
hFAS	TCGTGGGCTACAGCATGGT	GCCCTCTGAAGTCGAAGAAGAA
hSCD1	CCAGTCAACTCCTCGCACTT	AGCCAGGTTTGTAGTACCTCC
hPPARα	GGACAGCAAATCTTGAAGCAGC	CTCTGATCCCTCTAGCACCTT
hCPT1	TTTCCTTGCTGAGGTGCTCT	TCTCGCCTGCAATCATGTAG
hACO	TCCTGCCCACCTTGCTICAC	TTGGGGCCGATGTCACCAAC
hCAT	GAACTGTCCCTACCGTGCTCGA	CCAGAATATTGGATGCTGTGCTCCAGG
hSOD1	AATGGACCAGTGAAGGTGTGGGG	CACATTGCCCAAGTCTCCAACATGC
hGpx	CGGCCCAGTCGGTGTATGC	CGTGGTGCCTCAGAGGGAC
hTNFα	TGCTTGTTCCTCAGCCTCTT	ATGGGCTACAGGCTTGTCACT
hIL-6	ACTCACCTCTTCAGAACGAATTG	CCATCTTTGGAAGGTTCAGGTTG
hMCP1	CCCCAGTCACCTGCTGTTAT	TGGAATCCTGAACCCACTTC
hm18s	CGGCTACCACATCCAAGGAA	GCTGGAATTACCGCGGCT

Mouse

Gene	Forward primer	Reverse primer
mSREBP1c	GGAGCCATGGATTGCACATT	GGCCCGGGAAGTCACTGT
mCYP2E1	CTTTGCAGGAACAGAGACCA	ATGCACTACAGCGTCCATGT
mFAS	AGG TGG TGA TAG CCG GTA TGT	TGG GTA ATC CAT AGA GCC CAG
mSCD1	CTGTACGGGATCATACTGGTTC	GCCGTGCCTTGTAAGTTCTG
mPPARα	AGCTGGTGTAGCAAGTGT	TCTGCTTTCAGTTTTGCTTT
mCPT1	ACTCCTGGAAGAAGAAGTTCA	AGTATCTTTGACAGCTGGGAC
mACO	TGTTAAGAAGAGTGCCACCAT	ATCCATCTCTTCATAACCAAATTT
mCAT	TGAGAAGCCTAAGAACGCAATTC	\CCCTTCGCAGCCATGTG
mSOD1	CCAGTGCAGGACCTCATTTT	GTTTACTGCGCAATCCCAAT
mGpx1	CCACCGTGTATGCCTTCTCC	GATCGTGGTGCCTCAGAGAG
mTNFα	CCCTCACACTCAGATCATCTTCT	GCTACGACGTGGGCTACAG
mIL-6	TAGTCCTTCCTACCCCAATTTCC	TTGGTCCTTAGCCACTCCTTC
mMCP1	GCATCCACGTGTTGGCTCA	CTCCAGCCTACTCATTGGGATCA

mAdh1	TGAAGACGGTTACTG TCAAAGTGC	AGTGTGTGTGGCGGTTTTTCTC
Aldh2	GCAAAGCTGCGGTGCTAT	TCACACAAGTCACCCCTTCTC
hm18s	CGGCTACCACATCCAAGGAA	GCTGGAATTACCGCGGCT
